# Bidirectional Associations Between Engagement with Tobacco Information on Social Media and Young Adults’ Depressive Symptoms: A Longitudinal Cross-Lagged Analysis

**DOI:** 10.3390/bs16050653

**Published:** 2026-04-26

**Authors:** Qinghua Yang, C. Nathan Marti, Jacob E. Thomas, Alexandra Loukas

**Affiliations:** 1 Department of Communication Studies, Bob Schieffer College of Communication, Texas Christian University, Fort Worth, TX 76129, USA; 2 Department of Kinesiology and Health Education, College of Education, The University of Texas at Austin, Austin, TX 78712, USA; nate.marti@utexas.edu (C.N.M.); jacob.thomas@utexas.edu (J.E.T.); alexandra.loukas@austin.utexas.edu (A.L.)

**Keywords:** tobacco, electronic nicotine delivery systems, depression, young adults, social media

## Abstract

This study examined the cross-lagged associations between self-reported exposure to and engagement with tobacco/nicotine information on social media and depressive symptoms among young adults. Participants were 4267 20–32-year-olds (*M*_age_ 23.28, *SD* = 2.30 at baseline, 64.8% females) recruited in the Marketing and Promotions across Colleges in Texas study. Data for this study were collected in spring 2017 (baseline) and spring 2018 (follow-up). Results indicate that although self-reported exposure to and engagement with tobacco information, which were dichotomized, did not significantly predict subsequent depressive symptoms, young adults with clinically significant symptoms of depression were more likely than their peers to be exposed to (β = 0.10, *p* < 0.001) and engage with ($\beta_{pro}$ = 0.08, *p* < 0.01; $\beta_{anti}$ = 0.08, *p* < 0.05) tobacco information on social media one year later. The findings indicate that young adults with depressive symptoms may be susceptible to persuasive tobacco marketing on social media and in turn to potentially using tobacco and nicotine products. Findings call for regulation around tobacco marketing information on social media, especially for young adults with elevated depressive symptoms, a group at heightened risk for tobacco and ENDS use.

## 1. Introduction

Depression is one of the most prevalent mental health issues among young adults (Thapar et al., 2022). In a 2024 study, 57% of young adult college students reported moderate or severe depressive symptoms, among whom only 61% received mental health therapy or counseling (University of Michigan, 2024), while others may turn to social media for advice or self-medication. As the heaviest social media users (Auxier & Anderson, 2021), young adults are especially susceptible to persuasive normative and marketing messages, since they are undergoing cognitive development (Romer et al., 2017). Meta-analytic evidence indicates a positive association between elevated depressive symptoms and social media use (Cunningham et al., 2021; C. Huang, 2017; Yoon et al., 2019), such that young adults with elevated depressive symptoms are more likely than their peers to use and spend time on social media (C. Huang, 2017; Yoon et al., 2019). In addition, a longitudinal survey documented that excessive use of social media predicted subsequent depressive symptoms (Brailovskaia & Margraf, 2017). However, existing research is limited to examining only overall social media use, with a low level of granularity. Additional research is needed to pinpoint the association between depressive symptoms and specific content on social media, such as messages related to tobacco and nicotine products (hereafter referred to as tobacco).

The tobacco industry has leveraged social media to promote its products in past decades, but limited research examines the associations between depressive symptoms and exposure to or engagement with tobacco-related information on social media. *Exposure* refers to the passive viewing of tobacco-related information, whereas *engagement* indicates users’ active interaction with this content, such as liking, sharing, and posting tobacco-related information (Yang et al., 2024). In this study, we follow previous conceptualizations of health information exposure, defining tobacco/ENDS information exposure as information participants attend to and can subsequently recall with minimal prompting (Kelly et al., 2009; Niederdeppe et al., 2007). Moreover, no study has examined the direction of associations between exposure to and engagement with tobacco information on social media and young adults’ depressive symptoms longitudinally. Such gaps in the literature hinder our understanding of the bidirectional associations between exposure to and engagement with tobacco-related social media messages and depressive symptoms, and constrain tobacco regulation and intervention efforts targeting young adults with depression. To fill this gap, the current study aimed to identify the bidirectional associations between tobacco information exposure and engagement on social media and depressive symptoms among young adults across a one-year period. Our study not only contributes to the social media effects scholarship but also provides important implications for tobacco regulatory science.

### 1.1. Social Media Use and Depressive Symptoms Among Young Adults

A growing number of studies indicate a positive association between social media use, in general, and depressive symptoms (Feinstein et al., 2013; Lin et al., 2016; Primack et al., 2017; Yang et al., 2022). Social media use may be associated with depressive symptoms for a variety of reasons, including increased risk for negative social comparison with others (Feinstein et al., 2013) or increased exposure to cyber-bullying (Lin et al., 2016). Multiple meta-analyses have synthesized this body of scholarship, most reporting small but significant effect sizes between social media variables and depressive symptoms. For example, C. Huang (2017) reported a negative association between time spent on social media and overall psychological well-being. Yoon et al. (2019) found positive associations between depression and time spent on social media as well as social media checking frequency, which were consistent across age groups and genders. In the same vein, Cunningham et al. (2021) documented positive associations between depressive symptoms and time spent on social media, intensity of using social media, and problematic social media use, and pointed out the necessity of prospective, longitudinal research attempting to indicate the causal and transitional associations between social media use and depressive symptoms.

Despite scholarly attention on the association between social media use and depressive symptoms, most existing studies are cross-sectional, and none disentangle the directions of associations between tobacco information exposure and engagement on social media and depression (Cunningham et al., 2021). *Exposure* refers to an individual’s routine and accidental or passive acquisition of tobacco-related information on social media that has not been actively sought, whereas *engagement* is individuals’ active interaction with information, such as posting or responding to tobacco-related content on social media (Clendennen et al., 2020; Yang et al., 2023). Previous research indicates that passive social media use, which may trigger upward social comparison and lower self-esteem, is more consistently associated with users’ depressive symptoms than active use, which promotes social support and self-expression (Thorisdottir et al., 2019). Given this prior research, exposure to and engagement with tobacco information on social media may also be associated with depressive symptoms differently. To fill the gap in the literature, we focus on the bidirectional associations between depressive symptoms and young adults’ exposure to and engagement with tobacco information on social media across a one-year period. That is, we examine if depressive symptoms contribute to subsequent increases in tobacco information exposure and engagement on social media one year later, or vice versa, if exposure to and engagement with tobacco information contribute to increases in subsequent depressive symptoms one year later.

### 1.2. Young Adults’ Exposure to and Engagement with Tobacco Information on Social Media

Recent years have witnessed a shift in the promotion of tobacco, particularly nicotine products, such as electronic nicotine delivery systems (ENDS), from traditional media to social media (Ali et al., 2020). Young adults, as the heaviest social media users (Auxier & Anderson, 2021), are susceptible to not only peer influence of tobacco use (Schaefer et al., 2013) but also persuasive advertising messages because they may not be able to recognize the selling intent of well-produced advertising of ENDS products on social media (Biener & Albers, 2004), which are unregulated in the United States. ENDS use by young adults has substantially increased in the last few years and surpassed that of older adults in 2021 (Erhabor et al., 2023; Patrick et al., 2022). Cross-sectional and longitudinal evidence indicates that the rise in ENDS use and dependence among young adults is associated with their tobacco information exposure and engagement on social media (Clendennen et al., 2020; Hébert et al., 2017; Yang et al., 2023; Yang et al., 2024).

Although little is known about how or why exposure to and engagement with tobacco information on social media are associated with depressive symptoms, one cross-sectional study (Azagba et al., 2024) indicates that elevated depressive symptoms may mediate or account for the associations between general social media use and ENDS use. The study’s findings further suggest that social media use may contribute to subsequent depressive symptoms, but the cross-sectional nature precludes conclusions regarding temporal precedence, or the direction of associations. Additionally, tobacco use on social media is frequently presented in the context of social and festive gatherings (Liang et al., 2015). Such messages may result in upward social comparisons, leading to negative feelings, such as lower self-esteem and loneliness, which in turn may increase the risk of depression (Feinstein et al., 2013). Other social media content that discourages ENDS use by emphasizing the health risks of ENDS products (Lee et al., 2023) may heighten depressive symptoms by arousing fear and negative affect. On the other hand, a recent automated content analytic study reported that ENDS-related videos on TikTok also highlight the sense of belonging among ENDS users (Lee et al., 2024), which may lead to enhanced perceived social support and reduced depressive symptoms. Given the paucity of longitudinal research, the current study will use prospective data to determine if tobacco-related exposure and engagement on social media contribute to subsequent depressive symptoms. Thus, the first hypothesis (H1) was proposed as follows.

**H1:**  *Young adults’ (a) exposure to and (b) engagement with tobacco information on social media will predict their subsequent depressive symptoms one year later.*

### 1.3. Young Adults’ Depressive Symptoms and Social Media Exposure and Engagement

It is also possible, however, that depressive symptoms will contribute to subsequent tobacco-related social media exposure and engagement. Social media is an important platform for tobacco information seeking and experience sharing (Rutherford et al., 2023; Yang et al., 2018), particularly for young adults with depressive symptoms. Coping with stress and depression is one of the major themes of user-generated messages related to ENDS use on Twitter (Kim et al., 2023). User-generated messages on social media tend to glamorize substance use among young adults (Boyle et al., 2017), with vaping being depicted as an enjoyable behavior associated with fun, attractiveness, and social success (Zhan et al., 2017). Additionally, industry-generated ENDS promotional messages also glamorize tobacco use by framing vaping as success-oriented and cool (Alpert et al., 2019). As such, young adults with elevated depressive symptoms may be particularly attracted to these “double-dose” positive messages to cope with their negative affect. From this perspective, elevated levels of depressive symptoms may contribute to increased subsequent exposure to and engagement with tobacco-related social media messages. The second hypothesis was formulated as follows.

**H2:**  *Young adults’ depressive symptoms will increase their subsequent (a) exposure to and (b) engagement with tobacco information on social media one year later.*

By testing these two hypotheses, this study aims to extend the existing literature by examining the bidirectional associations between depressive symptoms and exposure to and engagement with tobacco-related social media among young adults. We focused on clinically significant depressive symptoms (Andresen et al., 1994) as the conceptual definition of depression. Furthermore, we distinguish between social media messages that are pro-tobacco, encouraging use (e.g., industry-sponsored promotional messages) or anti-tobacco, discouraging use (e.g., public health anti-smoking campaign messages). Considering that pro-tobacco and anti-tobacco engagement influences young adults’ ENDS use in opposite directions (Yang et al., 2023), they might impact subsequent depressive symptoms differently as well. For instance, exposure to and engagement with anti-tobacco social media content emphasizing the health threats of ENDS products (Lee et al., 2023) may increase participants’ risk of depressive symptoms, whereas pro-tobacco content highlighting normalization and social belonging of using ENDS (Lee et al., 2024) may alleviate stress and depressive symptoms. Thus, we examined both pro- and anti-tobacco engagement and their bi-directional associations with clinically significant depressive symptoms to generate a more nuanced understanding. We conducted cross-lagged path analyses to test hypotheses and to generate longitudinal evidence of the bi-directional associations.

## 2. Methods

### 2.1. Procedures and Participants

This study is a secondary data analysis of the Marketing and Promotions Across Colleges in Texas (M-PACT) study, which was approved by the university’s Institutional Review Board. Project M-PACT is a multi-wave surveillance study that followed a cohort of 5482 students from 12 two- and 12 four-year Texas colleges in the five counties surrounding Austin, Dallas/Fort Worth, Houston, and San Antonio to participate in a web-based survey from 2014 to 2019. The study analyzed marketing exposure, nicotine product use trajectories and transitions, and nicotine use exposure/engagement on social media platforms across 24 Texas campuses. To be eligible, participants were (1) 18–29 years old, and (2) full- or part-time degree- or certificate-seeking undergraduates attending a 4-year college or a vocational/technical program at a 2-year college. Detailed study procedures can be found elsewhere (Clendennen et al., 2020; Yang et al., 2023). Data in this study were from the spring 2017 (hereafter referred to as Time1 [T1] or baseline) and spring 2018 (one-year follow-up; Time 2 [T2]) data collection waves. Data from these waves were used because they captured the rapid increase in vape pod sales that occurred in late 2017 (J. Huang et al., 2019). Among the original M-PACT cohort, 80% participated in the baseline survey (*n* = 4384). In total, 4267 participants who completed both waves were included in the analyses, indicating a strong retention rate across the one-year period from baseline to follow-up (97.3%). The minimal attrition rate is a major methodological strength of the current study.

### 2.2. Measures

*Self-reported exposure to tobacco information on social media,* adopted from previous studies (Clendennen et al., 2020; Yang et al., 2023), was measured by asking participants to report how often they saw any advertisements for any tobacco and nicotine products on seven social media platforms (i.e., Facebook, Instagram, Twitter, Snapchat, YouTube, Pinterest, and Reddit) during the past 30 days at T1 and T2 on a 5-point Likert scale (1 = *never*, 5 = *very frequently*; $M_{T1}=$ 1.06, *SD* = 0.30; $M_{T2}=$ 1.07, *SD* = 0.33). These descriptive statistics indicate highly skewed distributions with means near the scale floor (1 = ‘Never’) and small SDs, reflecting minimal spread. According to MacCallum et al. (2002), dichotomization is legitimate and justified when distributions are highly skewed. As such, the items were recoded into dichotomous variables with 0 = *never exposed* and 1 = *ever exposed.* Given the low percentage of participants who had ever been exposed to tobacco and nicotine advertising on any specific social media platform (ranging from 4.7% on Pinterest at T1 to 14.8% on Facebook at T1), we aggregated these variables to be tobacco and nicotine advertising self-reported exposure on any social media platform (0 = *never exposed*, 1 = *ever exposed*).

*Self-reported engagement with tobacco information on social media* was measured by six items at T1 and T2. Three items queried about participants’ self-reported engagement with pro-tobacco information (e.g., posting thoughts or comments about the positive aspects of tobacco or ENDS use) and three about their self-reported engagement with anti-tobacco information (e.g., posting thoughts or comments about the negative aspects of tobacco or ENDS use). Participants were asked to rate how often they engaged with tobacco and nicotine products—i.e., (a) posting pro- or anti-tobacco links, (b) posting pro- or anti-tobacco thoughts or comments, and (c) encouraging or discouraging other people from using a tobacco and nicotine product—on social media in the past 30 days (Clendennen et al., 2020; Hébert et al., 2017). Items were coded on a 5-point Likert scale (1 = *never*, 5 = *very frequently*). The items demonstrated satisfactory reliability assessed using Cronbach’s α for both pro- (α = 0.82 at T1, α = 0.85 at T2; $M_{T1}=$ 1.17, *SD* = 0.44; $M_{T2}=$ 1.17, *SD* = 0.45) and anti-tobacco engagement (α = 0.76 at T1, α = 0.78 at T2; $M_{T1}=$ 1.12, *SD* = 0.38; $M_{T2}=$ 1.14, *SD* = 0.44). Two composite scores were created by summing the three items for pro-tobacco engagement and the three items for anti-tobacco engagement, respectively, with higher scores reflecting higher levels of engagement. Considering the low frequency of both pro- and anti-tobacco engagement (see Table 1), these variables were dichotomized (0 = *never engaged*, 1 = *ever engaged*).

*Covariates.* All models were adjusted for participants’ sociodemographic characteristics at baseline as potential confounders, including age, sex (0 = *male*, 1 = *female*), and race/ethnicity (i.e., non-Hispanic white, Hispanic or Latino, African American or black, Asian, and other). Participants’ race/ethnicity was dummy coded with other race/ethnicity as the reference group.

### 2.3. Analyses

Cross-lagged path analyses were performed to examine the bidirectional associations between self-reported exposure to and engagement with tobacco information on social media and depressive symptoms using a path model in *Mplus* 8. Although a non-significant $\chi^{2}$ test indicates an adequate model fit, other indices were also used to assess the model fit, because the $\chi^{2}$ value is almost always statistically significant when the sample size is over 400 (Bentler & Bonett, 1980). Specifically, a value smaller than 0.08 for the standardized root-mean-square residual (SRMR) and a value smaller than 0.05 for the root-mean square of approximation (RMSEA) suggest a reasonable fit (Kline, 2018). Values greater than 0.90 for the comparative fit index (CFI) and Tucker–Lewis Index (TLI) indicate a good fit (Kline, 2018). The model included four stability paths from all four T1 variables to T2 assessment of the same variables and six cross-lagged paths from T1 self-reported exposure and pro-/anti-engagement to T2 depressive symptoms and vice versa from T1 depressive symptoms to T2 self-reported exposure and pro-/anti-engagement. The model was also adjusted for demographic variables (i.e., age, sex, race/ethnicity) at T1 as potential confounders.

## 3. Results

### 3.1. Participants’ Characteristics and Substance Use

Participants were 23.28 years old on average (Min = 20.21, Max = 32.33, SD = 2.30) at T1. The majority of participants were females (63.8%) and self-reported as non-Hispanic White (35.7%) or Hispanic (30.8%). The prevalence of self-reported clinically significant depressive symptoms among participants was lower at T2 (29.4%) than at T1 (33.5%). Similarly, fewer participants (16.5%) reported being exposed to tobacco information on social media at T2, and more participants engaged with anti-tobacco information compared to pro-tobacco information at T1 and T2 (see Table 1 for details). The odds ratios indicate that differences between T1 and T2 for all variables were small in magnitude.

### 3.2. Cross-Lagged Analyses

**Figure 1:**
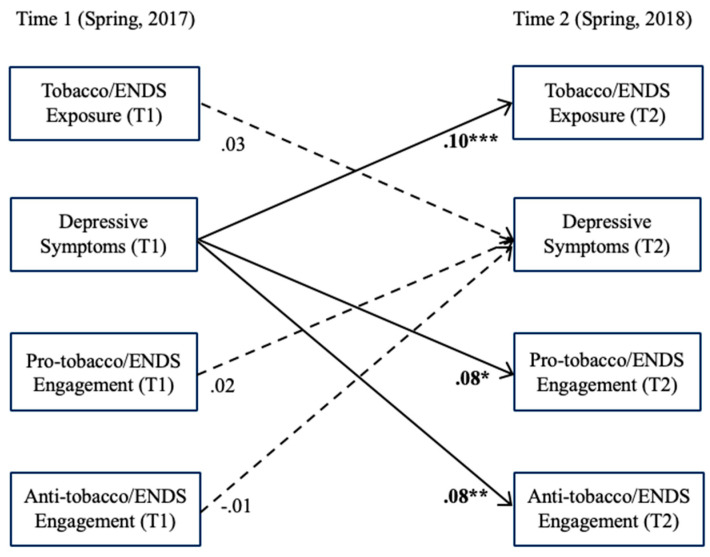
Cross-Lagged Model between Social Media Exposure/Engagement and Depressive Symptoms. *Note*. * *p* < 0.05, ** *p* < 0.01, *** *p* < 0.001. For visual clarity, the stability paths, which are all statistically significant and positive, and all covariates are not shown in this figure. Nonsignificant paths are represented by a dashed line.

The model fit the data well ($\chi^{2}$[26] = 87.24, *p* < 0.001; CFI = 0.96, TLI = 0.90, RMSEA = 0.023 [90% CIs 0.018–0.029], SRMR = 0.055) based on the model fit indices specified above. Stability paths from all T1 variables to the same T2 variables were all significant and positive. Results from cross-lagged analyses indicated that self-reported exposure to and engagement with pro- or anti-tobacco-related information on social media did not predict subsequent depressive symptoms, which failed to support H1a and H1b. On the other hand, however, depressive symptoms predicted a higher likelihood of self-reported exposure to (β = 0.10, SE = 0.02, *p* < 0.001; *OR* = 1.38) and engagement with pro- (β = 0.08, SE = 0.02, *p* < 0.01; *OR* = 1.29) and anti-tobacco (β = 0.08, SE = 0.03, *p* < 0.05; *OR* = 1.31) information on social media one year later, with small but significant effects, supporting both H2a and H2b (see Figure 1).

## 4. Discussion

Our cross-lagged analyses indicated small but significant effects, indicating that young adults with clinically significant symptoms of depression were more likely than their peers to be exposed to and engage with tobacco-related information on social media one year later, which is consistent with H2. However, we did not find support for the opposite direction of effects (H1); that is, we did not observe longitudinal associations between self-reported exposure to and engagement with tobacco-related on social media and subsequent depressive symptoms among young adults, despite meta-analytic evidence documenting a small but significant association between social media use and depressive symptoms (Cunningham et al., 2021; C. Huang, 2017; Yoon et al., 2019). The finding is also at odds with user-generated tobacco-related messages on social media that describe them as a coping mechanism for depressive symptoms (Kim et al., 2023). One possible explanation is that the null effect may be attributed to the diversity of tobacco-related information on social media. For instance, messages with high sensation value, which attract and appeal to receivers, especially higher sensation seekers (Noar et al., 2010), may lead to positive emotions and temporarily alleviate depressive symptoms, whereas other messages, including negative emotional appeals (e.g., guilt, fear), may increase the risk of depression (Kohn et al., 1982). Or, it is also plausible that although general social media use may lead to depression because of upward social comparison (Feinstein et al., 2013) and cyber-bullying (Lin et al., 2016), such mechanisms are not prominent with tobacco-specific information. However, given that these speculative mechanisms were not directly analyzed in the current study, more content-analytic and longitudinal research is needed to identify additional factors and mechanisms explaining whether and how young adults’ exposure to and engagement with tobacco-related information might influence their subsequent depressive symptoms.

The current findings establish a connection between antecedent depressive symptoms and later likelihood of being exposed to and engaged with tobacco-related information on social media. Existing evidence indicates that young adults with elevated depressive symptoms are more likely to use and be dependent on tobacco, including ENDS products, and to believe these products will alleviate their symptoms (Thomas et al., 2024; Truth Initiative, 2021). Thus, it is plausible that depressed young adults may self-medicate with tobacco and ENDS products, with the expectation that using these products could help alleviate stress or manage negative moods. Such a need for tobacco and ENDS products may impel depressed young adults to attend to and engage with tobacco-related information on social media, and the marketing of pro-tobacco information may further encourage them to use the products, forming a vicious cycle. On the other hand, young adults who self-medicate with tobacco and ENDS products may also consider terminating the use of tobacco products and therefore join quitting-related online support groups, which increases their likelihood of engaging with anti-tobacco information on social media. Considering that self-reported exposure to and engagement with tobacco-related social media information are associated with young adults’ subsequent vaping behaviors (Yang et al., 2023), those with more severe depressive symptoms may be at elevated risk of vaping because of that persuasive marketing information. Thus, pro-tobacco advertising exposure and engagement on social media could be a key mechanism linking depression and increased vaping behaviors among young adults. Given that tobacco-related information may function as an important coping mechanism for young adults with depressive symptoms, it is recommended that tobacco-related content be accompanied by reminders, such as warning of the risk of using tobacco products and providing the help lines by which they could seek support. Algorithm-based detection of social media users with depressive symptoms may provide promising opportunities to protect them from promotional tobacco information. However, this approach may also introduce ethical and privacy concerns, such as misuse of personal information, limited transparency, and possible misclassification of individuals as depressed when they are not, which may lead to unintended consequences (Jobin et al., 2019; Mikal et al., 2016). Thus, future tobacco regulatory efforts targeting populations with elevated depressive symptoms on social media should adhere to the five ethical principles, i.e., transparency, fairness, non-maleficence, responsibility, and privacy (Jobin et al., 2019) while developing classifiers.

### Limitations and Future Research

Our findings should be interpreted with caution. First, our sample, albeit demographically diverse, was college students recruited in Texas, which is not nationally representative and thus limits the generalizability of our findings. Second, our measures of all variables were based on self-reports, which, although a prevalent method used to assess young people’s engagement with tobacco-related information on social media and their depressive symptoms (Clendennen et al., 2020; Hébert et al., 2017; Yang et al., 2023), are subject to recall and desirability bias. For instance, participants in our study reported a higher level of engagement with anti-tobacco/ENDS information than pro-tobacco/ENDS information, which is at odds with the content analytic evidence documenting that pro-e-cigarette messages make up a large portion of e-cigarette-related content on social media (e.g., Laestadius et al., 2019a, 2019b; Yang et al., 2018). Such discrepancies may be attributed to participants’ difficulty in recalling their actual engagement with tobacco/ENDS information or their concerns of being judged negatively by self-reporting being engaged with e-cigarette promotional content. Given this, replications are needed with nationally representative samples and applying mixed methods combining subjective and objective measures to assess young adults’ tobacco-related social media engagement more comprehensively, such as ecological momentary assessment and automated textual analyses. Future research should also integrate objective digital trace data, such as social media platform logs or passive sensing of tobacco-related content exposure, with self-reported mental health measures to triangulate findings and reduce potential methodological bias. Third, the current model did not adjust for baseline tobacco/ENDS use behavior. While this was an intentional decision given that tobacco/ENDS use may itself be influenced by the social media exposure and engagement processes under study, future research should examine whether tobacco/ENDS use moderates or mediates these associations. Finally, due to the low occurrence of exposure and engagement, they were analyzed as dichotomized variables, which, while justified given the severely skewed distributions (MacCallum et al., 2002), has the risk of reducing variability, attenuating observed effect sizes, and obscuring potential dose–response relationships. Future research should reexamine our proposed bidirectional associations among young adults who report a higher level of exposure to and engagement with tobacco information on social media (e.g., young adults who are current users of ENDS products), operationalizing exposure and engagement as continuous variables.

## 5. Conclusions

Despite the limitations, the current study is among the first to disentangle the bidirectional relationships between tobacco-related social media use and depressive symptoms. Considering an interplay between depression, social media use, and smoking and vaping could signify an emerging behavioral health syndemic, our findings illuminate this interplay and provide guidance to tobacco regulatory science and public health by determining the longitudinal associations between depression and tobacco information exposure and engagement on social media, specifically among young adults. However, it should be noted that although statistically significant, the reported longitudinal effects are small in size and should be interpreted within a broader constellation of factors contributing to a behavioral health syndemic.

The study highlights the vulnerability of young adults with depressive symptoms, who are at higher risk of exposure to and engagement with tobacco-related information on social media. Such exposure and engagement may further increase their likelihood of vaping (Yang et al., 2023), since young adults may be ill-equipped to recognize the selling intent of meticulously crafted marketing of tobacco and ENDS products (Biener & Albers, 2004). Thus, our study calls for the need to regulate tobacco marketing information on social media, especially for young adults with elevated depressive symptoms as a vulnerable population. Considering the feasibility of detecting depressive symptoms with high accuracy based on users’ language and activity data on social media using machine learning approaches (Eichstaedt et al., 2018), social media platforms, for instance, could filter out tobacco and ENDS marketing and push algorithm-based anti-tobacco messages for those indicating clinically significant depressive symptomology.

## Figures and Tables

**Table 1:** Demographics for the sample in the cross-lagged model (N = 4267).

Variables	Baseline (Spring 2017)	Follow-Up (Spring 2018)	Effect Size
Age (years; *M*)	23.28 (*SD* = 2.30)		
Female (%)	64.8		
Race/ethnicity (%)			
Non-Hispanic White	35.7		
Hispanic	30.8		
Non-Hispanic African American	7.8		
Asian	18.1		
Other	7.5		
Depressive Symptoms (CES-D-10 ≥ 10; %)	33.5	29.4	*OR* = 1.21
Exposure to tobacco/ENDS information on social media (%)	20.5	16.5	*OR* = 1.30
Pro-engagement with tobacco/ENDS information on social media (%)	6.8	6.2	*OR* = 1.10
Anti-engagement with tobacco/ENDS information on social media (%)	18.8	16.2	*OR* = 1.20

*Note. M* = Mean. *SD* = Standard deviation. *OR* = Odds ratio. The percentage was calculated based on the final sample included in the cross-lagged model without including the cases with missing data. The last column shows the effect sizes comparing the percentage of T1 and T2 focal variables. *Depressive symptoms* were assessed at T1 and T2 using the 10-item short-form Center for Epidemiologic Studies Depression 10 Scale (CES-D-10) (Andresen et al., 1994). The CES-D-10 is a valid measure of depressive symptoms for young adults that includes ten items evaluating participants’ feelings in the past week, including eight assessing negative mood (e.g., “I felt lonely/fearful”) and two assessing positive mood (e.g., “I was happy/hopeful”). The response choices were given a score of 0 = *rarely or none of the time (0 days)* to 3 *= most or all of the time (5–7 days)*, with the two positive mood items reverse-scored (α = 0.85 at T1, α = 0.85 at T2). Scores were considered invalid if more than one item was missing. If only one item was missing, its value was imputed as the mean of the participant’s other nine-item scores. Items were summed and total scores ranged from 0 to 30. A score of 10 or greater was considered as screening positive for clinically significant depressive symptomology (Andresen et al., 1994). Thus, the total score was dichotomized (0 = *CES-D-10* < 10, 1 = *CES-D*-10 ≥ 10) and participants with a total score of 10 or greater were considered to have clinically significant depressive symptoms. Dichotomizing the CES-D-10 score at the cut-off point of 10 is a validated and widely applied operationalization of depressive symptoms among young adults (e.g., Fowler-Brown et al., 2012; Loukas et al., 2023).

## Data Availability

The data are available upon appropriate request.

## References

[B1-behavsci-16-00653] Ali F. R. M., Marynak K. L., Kim Y., Binns S., Emery S. L., Gomez Y., King B. A. (2020). E-cigarette advertising expenditures in the United States, 2014–2018. Tobacco Control.

[B2-behavsci-16-00653] Alpert J. M., Jaisle A., Chen H. (2019). A content analysis of the promotional strategies employed by e-cigarette brands on Twitter. Health Marketing Quarterly.

[B3-behavsci-16-00653] Andresen E. M., Malmgren J. A., Carter W. B., Patrick D. L. (1994). Screening for depression in well older adults: Evaluation of a short form of the CES-D (Center for Epidemiologic Studies Depression Scale). American Journal of Preventive Medicine.

[B4-behavsci-16-00653] Auxier B., Anderson M. (2021). Social media use in 2021. Pew Research Center.

[B5-behavsci-16-00653] Azagba S., Ebling T., Korkmaz A. (2024). Social media and e-cigarette use: The mediating role of mental health conditions. Journal of Affective Disorders.

[B6-behavsci-16-00653] Bentler P. M., Bonett D. G. (1980). Significance tests and goodness of fit in the analysis of covariance structures. Psychological Bulletin.

[B7-behavsci-16-00653] Biener L., Albers A. B. (2004). Young adults: Vulnerable new targets of tobacco marketing. American Journal of Public Health.

[B8-behavsci-16-00653] Boyle S. C., Earle A. M., LaBrie J. W., Ballou K. (2017). Facebook dethroned: Revealing the more likely social media destinations for college students’ depictions of underage drinking. Addictive Behaviors.

[B9-behavsci-16-00653] Brailovskaia J., Margraf J. (2017). Facebook Addiction Disorder (FAD) among German students—A longitudinal approach. PLoS ONE.

[B10-behavsci-16-00653] Clendennen S. L., Loukas A., Vandewater E. A., Perry C. L., Wilkinson A. V. (2020). Exposure and engagement with tobacco-related social media and associations with subsequent tobacco use among young adults: A longitudinal analysis. Drug and Alcohol Dependence.

[B11-behavsci-16-00653] Cunningham S., Hudson C. C., Harkness K. (2021). Social media and depression symptoms: A meta-analysis. Research on Child and Adolescent Psychopathology.

[B12-behavsci-16-00653] Eichstaedt J. C., Smith R. J., Merchant R. M., Ungar L. H., Crutchley P., Preoţiuc-Pietro D., Asch D. A., Schwartz H. A. (2018). Facebook language predicts depression in medical records. Proceedings of the National Academy of Sciences.

[B13-behavsci-16-00653] Erhabor J., Boakye E., Obisesan O., Osei A. D., Tasdighi E., Mirbolouk H., DeFilippis A. P., Stokes A. C., Hirsch G. A., Benjamin E. J., Rodriguez C. J., El Shahawy O., Robertson R. M., Bhatnagar A., Blaha M. J. (2023). E-cigarette use among US adults in the 2021 behavioral risk factor surveillance system survey. JAMA Network Open.

[B14-behavsci-16-00653] Feinstein B. A., Hershenberg R., Bhatia V., Latack J. A., Meuwly N., Davila J. (2013). Negative social comparison on facebook and depressive symptoms: Rumination as a mechanism. Psychology of Popular Media Culture.

[B15-behavsci-16-00653] Fowler-Brown A. G., Ngo L. H., Wee C. C. (2012). The relationship between symptoms of depression and body weight in younger adults. Obesity.

[B16-behavsci-16-00653] Hébert E. T., Case K. R., Kelder S. H., Delk J., Perry C. L., Harrell M. B. (2017). Exposure and engagement with tobacco- and e-cigarette–related social media. Journal of Adolescent Health.

[B17-behavsci-16-00653] Huang C. (2017). Time spent on social network sites and psychological well-being: A meta-analysis. Cyberpsychology, Behavior and Social Networking.

[B18-behavsci-16-00653] Huang J., Duan Z., Kwok J., Binns S., Vera L. E., Kim Y., Szczypka G., Emery S. L. (2019). Vaping versus JUULing: How the extraordinary growth and marketing of JUUL transformed the US retail e-cigarette market. Tobacco Control.

[B19-behavsci-16-00653] Jobin A., Ienca M., Vayena E. (2019). The global landscape of AI ethics guidelines. Nature Machine Intelligence.

[B20-behavsci-16-00653] Kelly B. J., Niederdeppe J., Hornik R. C. (2009). Validating measures of scanned information exposure in the context of cancer prevention and screening behaviors. Journal of Health Communication.

[B21-behavsci-16-00653] Kim I., Begay C., Ma H. J., Orozco F. R., Rogers C. J., Valente T. W., Unger J. B. (2023). E-cigarette-related health beliefs expressed on Twitter within the U.S. AJPM Focus.

[B22-behavsci-16-00653] Kline R. B. (2018). Principles and practice of structural equation modeling.

[B23-behavsci-16-00653] Kohn P. M., Goodstadt M. S., Cook G. M., Sheppard M., Chan G. (1982). Ineffectiveness of threat appeals about drinking and driving. Accident Analysis and Prevention.

[B24-behavsci-16-00653] Laestadius L. I., Penndorf K. E., Seidl M., Cho Y. I. (2019a). Assessing the appeal of Instagram electronic cigarette refill liquid promotions and warnings among young adults: Mixed Methods Focus Group Study. Journal of Medical Internet Research.

[B25-behavsci-16-00653] Laestadius L. I., Wahl M. M., Pokhrel P., Cho Y. I. (2019b). From Apple to Werewolf: A content analysis of marketing for e-liquids on Instagram. Addictive Behaviors.

[B26-behavsci-16-00653] Lee J., Murthy D., Kong G. (2023). Content analysis of YouTube videos related to E-cigarettes and COVID-19. medRxiv.

[B27-behavsci-16-00653] Lee J., Ouellette R. R., Murthy D., Pretzer B., Anand T., Kong G. (2024). Identifying E-cigarette content on TikTok: Using a BERTopic modeling approach. Nicotine & Tobacco Research.

[B28-behavsci-16-00653] Liang Y., Zheng X., Zeng D. D., Zhou X., Leischow S. J., Chung W. (2015). Exploring how the tobacco industry presents and promotes itself in social media. Journal of Medical Internet Research.

[B29-behavsci-16-00653] Lin L. Y., Sidani J. E., Shensa A., Radovic A., Miller E., Colditz J. B., Hoffman B. L., Giles L. M., Primack B. A. (2016). Association between social media and depression among U.S. young adults. Depression and Anxiety.

[B30-behavsci-16-00653] Loukas A., Li X., Wilkinson A. V., Marti C. N. (2023). Longitudinal examination of ENDS use among young adult college students: Associations with depressive symptoms and sensation seeking. Prevention Science.

[B31-behavsci-16-00653] MacCallum R. C., Zhang S., Preacher K. J., Rucker D. D. (2002). On the practice of dichotomization of quantitative variables. Psychological Methods.

[B32-behavsci-16-00653] Mikal J., Hurst S., Conway M. (2016). Ethical issues in using Twitter for population-level depression monitoring: A qualitative study. BMC Medical Ethics.

[B33-behavsci-16-00653] Niederdeppe J., Hornik R. C., Kelly B. J., Frosch D. L., Romantan A., Stevens R. S., Barg F. K., Weiner J. L., Schwartz J. S. (2007). Examining the dimensions of cancer-related information seeking and scanning behavior. Health Communication.

[B34-behavsci-16-00653] Noar S. M., Palmgreen P., Zimmerman R. S., Lustria M. L. A., Lu H.-Y. (2010). Assessing the relationship between perceived message sensation value and perceived message effectiveness: Analysis of PSAs from an effective campaign. Communication Studies.

[B35-behavsci-16-00653] Patrick M. E., Schulenberg J. E., Miech R. A., Johnston L. D., O’Malley P. M., Bachman J. G., Institute for Social Research, University of Michigan (2022). Monitoring the future panel study annual report: National data on substance use among adults ages 19 to 60, 1976–2021.

[B36-behavsci-16-00653] Primack B. A., Shensa A., Escobar-Viera C. G., Barrett E. L., Sidani J. E., Colditz J. B., James A. E. (2017). Use of multiple social media platforms and symptoms of depression and anxiety: A nationally-representative study among U.S. young adults. Computers in Human Behavior.

[B37-behavsci-16-00653] Romer D., Reyna V. F., Satterthwaite T. D. (2017). Beyond stereotypes of adolescent risk taking: Placing the adolescent brain in developmental context. Developmental Cognitive Neuroscience.

[B38-behavsci-16-00653] Rutherford B. N., Lim C. C. W., Johnson B., Cheng B., Chung J., Huang S., Sun T., Leung J., Stjepanović D., Chan G. C. K. (2023). TurntTrending: A systematic review of substance use portrayals on social media platforms. Addiction.

[B39-behavsci-16-00653] Schaefer D. R., Adams J., Haas S. A. (2013). Social networks and smoking: Exploring the effects of peer influence and smoker popularity through simulations. Health Education & Behavior.

[B40-behavsci-16-00653] Thapar A., Eyre O., Patel V., Brent D. (2022). Depression in young people. The Lancet.

[B41-behavsci-16-00653] Thomas J. E., Pasch K. E., Nathan Marti C., Loukas A. (2024). Depressive symptoms prospectively increase risk for new onset cigarette and ENDS dependence symptoms. Addictive Behaviors.

[B42-behavsci-16-00653] Thorisdottir I. E., Sigurvinsdottir R., Asgeirsdottir B. B., Allegrante J. P., Sigfusdottir I. D. (2019). Active and passive social media use and symptoms of anxiety and depressed mood among Icelandic adolescents. Cyberpsychology, Behavior, and Social Networking.

[B43-behavsci-16-00653] Truth Initiative (2021). Many young people turn to nicotine to deal with stress, anxiety and depression, but don’t know it may be making them feel worse.

[B44-behavsci-16-00653] University of Michigan (2024). College students’ mental health improving, more finding support.

[B45-behavsci-16-00653] Yang Q., Clendennen S. L., Loukas A. (2023). How does social media exposure and engagement influence college students’ use of ENDS products? A cross-lagged longitudinal study. Health Communication.

[B46-behavsci-16-00653] Yang Q., Clendennen S. L., Marti N. C., Loukas A. (2024). Associations between social media engagement and young adults’ subsequent onset of ENDS dependence symptoms one year later. Addictive Behaviors.

[B47-behavsci-16-00653] Yang Q., Liu J., Rui J. (2022). Association between social network sites use and mental illness: A meta-analysis. Cyberpsychology.

[B48-behavsci-16-00653] Yang Q., Sangalang A., Rooney M., Maloney E., Emery S., Cappella J. N. (2018). How is marijuana vaping portrayed on YouTube? Content, features, popularity and retransmission of vaping marijuana YouTube videos. Journal of Health Communication.

[B49-behavsci-16-00653] Yoon S., Kleinman M., Mertz J., Brannick M. (2019). Is social network site usage related to depression? A meta-analysis of Facebook–depression relations. Journal of Affective Disorders.

[B50-behavsci-16-00653] Zhan Y., Liu R., Li Q., Leischow S. J., Zeng D. D. (2017). Identifying topics for e-cigarette user-generated contents: A case study from multiple social media platforms. Journal of Medical Internet Research.

